# The BoCML37-BoABA2 module positively regulates water-deficit tolerance in cabbage

**DOI:** 10.1093/plphys/kiaf610

**Published:** 2025-12-04

**Authors:** Jialei Ji, Yiwei Liu, Caihong Wang, Ying Li, Yue Zhang, Linqian Kuang, Wenxue Cao, Honghao Lv, Yong Wang, Limei Yang, Mu Zhuang, Yangyong Zhang

**Affiliations:** State Key Laboratory of Vegetable Biobreeding, Institute of Vegetables and Flowers, Chinese Academy of Agricultural Sciences, Beijing 100081, China; China Vegetable Seed Technology Co. Ltd. (Chongqing), Chongqing 402561, China; Administration and Managemengt Institute, Ministry of Agriculture and Rural Affairs, Beijing 102208, China; State Key Laboratory of Vegetable Biobreeding, Institute of Vegetables and Flowers, Chinese Academy of Agricultural Sciences, Beijing 100081, China; State Key Laboratory of Vegetable Biobreeding, Institute of Vegetables and Flowers, Chinese Academy of Agricultural Sciences, Beijing 100081, China; State Key Laboratory of Vegetable Biobreeding, Institute of Vegetables and Flowers, Chinese Academy of Agricultural Sciences, Beijing 100081, China; State Key Laboratory of Vegetable Biobreeding, Institute of Vegetables and Flowers, Chinese Academy of Agricultural Sciences, Beijing 100081, China; State Key Laboratory of Vegetable Biobreeding, Institute of Vegetables and Flowers, Chinese Academy of Agricultural Sciences, Beijing 100081, China; State Key Laboratory of Vegetable Biobreeding, Institute of Vegetables and Flowers, Chinese Academy of Agricultural Sciences, Beijing 100081, China; State Key Laboratory of Vegetable Biobreeding, Institute of Vegetables and Flowers, Chinese Academy of Agricultural Sciences, Beijing 100081, China; State Key Laboratory of Vegetable Biobreeding, Institute of Vegetables and Flowers, Chinese Academy of Agricultural Sciences, Beijing 100081, China; State Key Laboratory of Vegetable Biobreeding, Institute of Vegetables and Flowers, Chinese Academy of Agricultural Sciences, Beijing 100081, China; State Key Laboratory of Vegetable Biobreeding, Institute of Vegetables and Flowers, Chinese Academy of Agricultural Sciences, Beijing 100081, China

## Abstract

The calmodulin-like protein BoCML37 in cabbage directly activates the key enzyme BoABA2 for abscisic acid production, boosting drought tolerance and offering a target for breeding resilient crops.

Dear Editor,

The intensification of global warming has increased the frequency, duration, and geographical scope of droughts, posing severe threats to agriculture. As a vital cruciferous crop, cabbage (*Brassica oleracea* var. *capitata*) occupied 2.36 million hectares globally in 2023 (FAO, 2025), yet faces escalating drought/water-deficit impacts. This urgency demands innovative strategies, including deciphering molecular mechanisms of water-deficit (WD) tolerance, to develop resilient cabbage germplasm and cultivars.

Central to the WD response of plant are Ca^2+^ signals. Under WD stress, intracellular Ca^2+^ concentration in plants dramatically increases, generating spatiotemporally specific Ca^2+^ signals (Edel et al. 2017; Gong et al. 2020). These signals are perceived by plant-specific sensors like Calmodulin-like proteins (CMLs), which contain EF-hand domains for Ca^2+^ binding. They function analogously to calmodulin: binding target proteins upon calcium signal perception to modify their activity (Li et al. 2023). Although numerous CMLs linked to WD and other abiotic stresses are identified, their regulatory mechanisms remain poorly understood, largely due to the scarcity of identified functional target proteins. A prominent example is Arabidopsis *AtCML37*, which enhances WD tolerance by regulating abscisic acid (ABA) biosynthesis; however, its precise molecular mechanism and direct downstream targets remain elusive (Scholz et al. 2015; Heyer et al. 2022).

To bridge this critical knowledge gap and explore the potential of CML-mediated WD tolerance in cabbage, we focused on orthologs of key regulators like *AtCML37*. Building on our previous identification of 75 *BoCML* genes in cabbage (Wu et al. 2025), we analyzed their transcript abundance under WD stress (Supplementary Table S1). This revealed that *BoCML37* (*BolC09g022750.2J*), the sole direct ortholog of *AtCML37* in cabbage, is strikingly upregulated. It exhibited a remarkable 58.8-fold increase in shoots and a 4.7-fold increase in roots following WD treatment (Supplementary Fig. S1). This robust induction strongly positions *BoCML37* as a key mediator of WD stress responses in cabbage and a prime candidate for elucidating the mechanistic basis of CML37 function.

BoCML37 shares 82.2% sequence identity with AtCML37, with particularly high similarity of three EF-hand motifs (Supplementary Fig. S2). To functionally characterize *BoCML37*, we therefore generated three overexpression lines (CML-OE1, CML-OE2, CML-OE3) and two knockout lines (cml-CR1, cml-CR2) (Fig. 1A, Supplementary S3). Following two weeks of WD, the overexpression lines exhibited significantly enhanced tolerance, achieving survival rates approximately 90% higher than those of wild-type (WT) plants. In contrast, the knockout lines displayed increased WD sensitivity, with survival rates approximately 76% lower than the WT (Fig. 1B, Supplementary Table S2). Endogenous hormone analysis further revealed that under WD stress, ABA levels in the overexpression lines increased by 23% relative to the WT, whereas levels in the knockout lines decreased by 72% relative to the WT (Fig. 1C). Collectively, these results demonstrate that *BoCML37* positively regulates WD tolerance in cabbage, likely through modulating ABA biosynthesis. This functional conservation aligns with previous findings for *AtCML37*.

**Figure 1.**
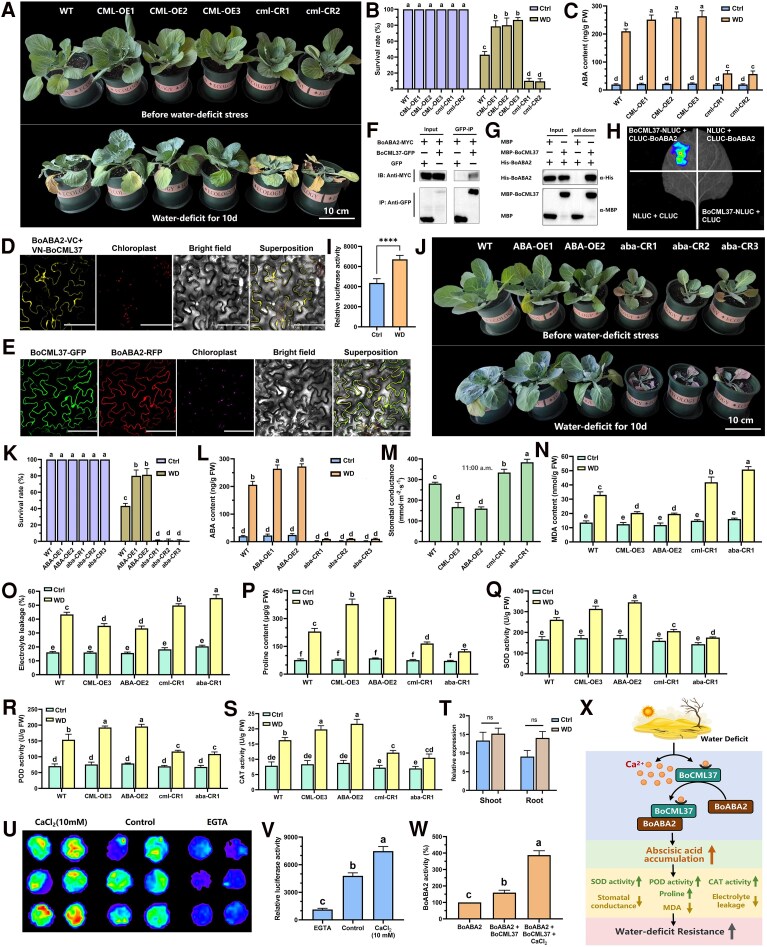
Functional analysis of the BoCML37-BoABA2 module in cabbage. **A)** The phenotypes of *BoCML37* overexpression lines (CML-OE1, CML-OE2, CML-OE3), knockout lines (cml-CR1, cml-CR2), and wild-type (WT) before and after a 10-day water-deficit (WD) treatment. Images were digitally extracted for comparison. **B)** The survival rate of *BoCML37* overexpression lines, knockout lines, and WT after two weeks of WD. Values represent means ± SE (n = 3). Different letters above bars denote significant differences (*P* < 0.05) as determined by Duncan's test. **C)** The ABA content in *BoCML37* overexpression lines, knockout lines, and WT under control (Ctrl) and 5-days-WD condition. Values represent means ± SE (n = 6). Different letters above bars denote significant differences (*P* < 0.05) as determined by Duncan's test. **D)** Analysis of the interaction between BoCML37 and BoABA2 using BiFC assay in *Nicotiana benthamiana*. Scale bar = 100 *μ*m. **E)** Subcellular localization of BoCML37 and BoABA2 in *N. benthamiana*. Scale bar = 100 *μ*m. **F-H)** Verification of the BoCML37-BoABA2 interaction using Co-IP (F), Pull-down (G), and Luciferase Complementation Assays (LCA) (H). **I)** 5-day-WD enhances BoCML37-BoABA2 interaction (LCA in *N. benthamiana*). Statistical significance was assessed by *t*-test. **** *P* < 0.0001. **J)** The phenotypic characteristics of *BoABA2* overexpression lines (ABA-OE1, ABA-OE2), knockout lines (aba-CR1, aba-CR2, aba-CR3), and WT before and after a 10-day WD treatment. Images were digitally extracted for comparison. **K)** The survival rate of *BoABA2* overexpression lines, knockout lines, and WT after two weeks of WD. Values represent means ± SE (n = 3). Different letters above bars denote significant differences (*P* < 0.05) as determined by Duncan's test. **L)** The ABA content in *BoABA2* overexpression/knockout lines and WT under control and 5-days-WD condition. Values represent means ± SE (n = 6). Different letters above bars denote significant differences (*P* < 0.05) as determined by Duncan's test. **M)** Stomatal conductance of transgenic lines measured at 11:00 AM. Values represent means ± SE (n = 6). Different letters above bars denote significant differences (*P* < 0.05) as determined by Duncan's test. **N-S)** MDA content (N), electrolyte leakage (O), proline content (P), SOD activity (Q), POD activity (R), and CAT activity (S) of transgenic lines before and 5 days after WD treatment. Values represent means ± SE (n = 6). Different letters above bars denote significant differences (*P* < 0.05) as determined by Duncan's test. **T)** The relative expression levels of *BoABA2* in shoots and roots of cabbage before and 5 days after WD treatment. Statistical significance was assessed by *t*-test. ns, non-significant, *P* > 0.05. **U**, **V)** The Ca^2+^-dependent interaction between BoCML37 and BoABA2 was analyzed in *N. benthamiana* leaves co-transformed with Agrobacterium mixtures harboring 35S::cLUC-BoABA2 and 35S::BoCML37-nLUC, which were incubated on 0.8% agar plates containing 10 mm CaCl_2_, 20 mm EGTA, or no supplement (control) for 48 h prior to luminescence imaging (U) and measuring the fluorescence intensity as the relative LUC activity (V). Images were digitally extracted for comparison. Values are presented as means ± SE (n = 6 leaf discs). Different letters above bars denote significant differences (*P* < 0.05) among treatments based on Duncan's test. **W)** BoCML37 binding enhances BoABA2 enzymatic activity *in vitro*. The activity of BoABA2 was determined as described in Supplementary Materials and Methods. Values represent means ± SE (n = 3). Different letters above bars denote significant differences (P < 0.05) as determined by Duncan's test. **X)** Regulatory pathway diagram of the BoCML37-BoABA2 module in WD resistance of cabbage.

To explore the potential mechanism by which BoCML37 modulates ABA biosynthesis, we first examined its subcellular localization. Fluorescence analysis revealed that BoCML37 is localized in the cytoplasm (Supplementary Fig. S4). Given that the primary sites of ABA biosynthesis in plants are plastids and the cytoplasm (Chen et al. 2020), this localization pattern prompted us to hypothesize that BoCML37 might specifically regulate the cytoplasmic steps of ABA biosynthesis. The cytoplasmic phase of ABA biosynthesis commences with the export of xanthoxin (synthesized in plastids) into the cytoplasm (Supplementary Fig. S5A). Here, xanthoxin dehydrogenase (ABA2) first catalyzes the conversion of xanthoxin to abscisic aldehyde, which is subsequently oxidized by abscisic-aldehyde oxidase (AAO3) to yield ABA (Supplementary Fig. S5). Therefore, ABA2 and AAO3 are cytoplasm-localized key enzymes in this pathway.

Based on homology analysis, we identified and cloned the cabbage homologs of *ABA2* (*BolC06g007060.2J*, designated *BoABA2*) and *AAO3* (*BolC04g053960.2J*, designated *BoAAO3*). Bimolecular fluorescence complementation (BiFC) assays revealed a specific interaction signal between BoCML37 and BoABA2 in the cytoplasm, while no interaction was detected between BoCML37 and BoAAO3 (Fig. 1D, Supplementary S6). Subcellular localization analysis further confirmed the overlapping localization of BoCML37 and BoABA2, supporting the notion that they function within the same subcellular compartment (Fig. 1E). This interaction was robustly validated through multiple independent methods: yeast two-hybrid, co-immunoprecipitation, pull-down assays, and luciferase complementation assays (LCA) all consistently demonstrated the direct binding between BoCML37 and BoABA2 (Fig. 1, F to H, Supplementary S7–S11). Interestingly, LCA revealed that 5-day-WD stress enhances BoCML37-BoABA2 interaction (Fig. 1I), suggesting that the WD-induced elevation in cytosolic calcium levels may further promote this interaction.

To further validate the function of BoABA2, we generated BoABA2-overexpressing lines (ABA-OE1 and ABA-OE2) and knockout lines (aba-CR1, aba-CR2, and aba-CR3) (Fig. 1J, Supplementary S12 and S13). Following two weeks of WD treatment, overexpression lines exhibited significantly enhanced WD tolerance, achieving survival rates approximately 86% higher than that of WT (Fig. 1, J and K). Conversely, knockout lines displayed growth retardation, dwarfism, compromised reproductive capacity, and extreme WD sensitivity, with nearly all plants perishing (survival rates <2%) under identical conditions (Supplementary Table S2S3). Endogenous hormone assays further confirmed that ABA levels in BoABA2-overexpressing lines rose markedly under WD stress, exceeding those in WT plants by 30% (Fig. 1L). In stark contrast, ABA synthesis was severely impaired in knockout lines: even under drought conditions, their ABA content remained below 11 *μ*g/g FW (approximately 5% of that in the WT) (Fig. 1L). Collectively, these genetic and biochemical data demonstrate that BoABA2 is indispensable for ABA biosynthesis and WD resistance in cabbage.

Given that ABA-mediated stress resistance triggers coordinated physiological adaptations, we examined key stress-response indicators in transgenic lines. *BoCML37*/*BoABA2*-overexpressing lines exhibited significantly reduced stomatal conductance, MDA content, and electrolyte leakage alongside markedly elevated proline content, SOD, POD, and CAT activities (Fig. 1M-S). These shifts—reduced oxidative damage markers and enhanced antioxidant capacity—represent hallmark protective responses to, to D stress (Qiu et al. 2023; Dong et al. 2024). Conversely, *BoCML37*/*BoABA2* knockout lines showed opposite trends across all parameters, confirming their hypersensitivity.

Notably, despite the critical role of *BoABA2* in enhancing WD tolerance, its transcript abundance showed no significant change under 5-day-WD treatment (Fig. 1T). This contrasts sharply with the robust WD-induced upregulation observed for key ABA biosynthesis genes *BoBCH*, *BoZEP*, *BoNCED3,* and *BoAAO3* (Supplementary Fig. S5), highlighting a unique regulatory pattern for BoABA2. This apparent paradox suggests that cabbage enhances WD tolerance primarily through post-translational activation of BoABA2—mediated by WD-induced BoCML37 and Ca^2+^ signal—rather than transcript abundance upregulation of *BoABA2* itself.

Given that the AtCML37 functions as a Ca^2+^ sensor (Scholz et al. 2014), we investigated whether Ca^2+^ regulates the BoCML37-BoABA2 interaction. LCA with CaCl_2_ or EGTA supplementation showed intensified BoCML37-BoABA2 interaction signals in CaCl_2_-treated leaves (similar to WD stress) but diminished signals in EGTA-treated leaves relative to controls, demonstrating strict Ca^2+^ dependence for complex formation (Figure 1, U and V).

To assess the functional consequence of this interaction, we measured BoABA2's catalytic activity in converting xanthoxin to abscisic aldehyde. Strikingly, when co-incubated with BoCML37 in the presence of Ca^2+^, BoABA2 activity increased by 3.88-fold relative to BoABA2 alone (Fig. 1W). This Ca^2+^-potentiated activation establishes a mechanistic link whereby WD-induced BoCML37 enhances ABA biosynthesis through direct stimulation of BoABA2 enzymatic function.

Collectively, our results delineate a complete molecular pathway for WD adaptation in cabbage centered on the BoCML37-BoABA2 module (Fig. 1X). Upon WD perception, elevated cytosolic Ca^2+^ acts as a secondary messenger while WD stress simultaneously upregulates transcript abundance of *BoCML37*. The resulting Ca^2+^-bound BoCML37 protein then directly binds to BoABA2, enhancing its catalytic activity in converting xanthoxin to abscisic aldehyde. This post-translational activation drives ABA biosynthesis, triggering downstream physiological adaptations—including stomatal closure, antioxidant activation, and osmolyte accumulation—that collectively enhance WD tolerance. These findings establish *BoCML37* and *BoABA2* as important targets for molecular breeding of WD-resilient cabbage cultivars.

## Supplementary Material

kiaf610_Supplementary_Data

## Data Availability

All data generated or analyzed in this study are included in this article and its Supplementary material.

## Supplementary data

The following materials are available in the online version of this article.

Supplementary Materials and Methods.

**Supplementary Figure S1.** Transcript abundance analysis of BoCML genes pre- and post-water-deficit treatment.

**Supplementary Figure S2.** Phylogenetic analysis of BoCML proteins and multiple sequence alignment of BoCML37 homologous proteins.

**Supplementary Figure S3.** Transcript abundance analysis of *BoCML37* in overexpression lines and variant analysis of *BoCML37* in gene-edited lines.

**Supplementary Figure S4.** Subcellular localization of the BoCML37 protein in *N. benthamiana* leaves.

**Supplementary Figure S5.** Transcript abundance analysis of ABA biosynthesis-related genes and subcellular localization of BoNCED3 (BolC01g048310.2J).

**Supplementary Figure S6.** Analysis of the interaction between BoCML37 and BoABA2/BoAAO3 by Bimolecular Fluorescence Complementation (BiFC) in *N. benthamiana*.

**Supplementary Figure S7.** Verification of the interaction between BoCML37 and BoABA2 by Yeast Two-Hybrid (Y2H) assay.

**Supplementary Figure S8.** SDS-PAGE of purified BoCML37 protein.

**Supplementary Figure S9.** SDS-PAGE of purified BoABA2 protein.

**Supplementary Figure S10.** Western blot of the co-immunoprecipitation (Co-IP) assay validating the interaction between BoCML37 and BoABA2.

**Supplementary Figure S11.** Western blot of the GST pull-down assay validating the interaction between BoCML37 and BoABA2.

**Supplementary Figure S12.** Phylogenetic analysis of BoABA2.

**Supplementary Figure S13.** Transcript abundance analysis of *BoABA2* in overexpression lines and variant analysis of *BoABA2* in gene-edited lines.

**Supplementary Table S1.** The primers used in this study.

**Supplementary Table S2.** The survival rate of the transgenic lines.

**Supplementary Table S3.** The reproductive capacity of the transgenic lines.

## Dive Curated Terms

The following phenotypic, genotypic, and functional terms are of significance to the work described in this paper:

AAO3 Gramene: AT2G27150
AAO3 Araport: AT2G27150

ABA2 Gramene: AT1G52340
ABA2 Araport: AT1G52340

CML37 Gramene: AT5G42380
CML37 Araport: AT5G42380
