# Supplementary material for: The BoCML37-BoABA2 module positively regulates water-deficit tolerance in cabbage
Source: Plant Physiol. 2025 Dec 4;199(4):kiaf610. doi: 10.1093/plphys/kiaf610 (PMC12677907; doi:10.1093/plphys/kiaf610)
Supplement: kiaf610_Supplementary_Data [file kiaf610_supplementary_data.pdf]

The BoCML37-BoABA2 module positively regulate water-deficit tolerance in cabbage

Jialei Ji[1,3,*], Yiwei Liu[2], Caihong Wang[1], Ying Li[1], Yue Zhang[1], Linqian Kuang[1], Wenxue Cao[1], Honghao Lv[1], Yong Wang[1], Limei Yang[1], Mu Zhuang[1], Yangyong Zhang[1,*]

[1]State Key Laboratory of Vegetable Biobreeding, Institute of Vegetables and Flowers, Chinese Academy of Agricultural Sciences, Beijing 100081, China.

[2]Agricultural Management Institute, Ministry of Agriculture and Rural Affairs, Beijing 102208, China

[3]China Vegetable Seed Technology Co. Ltd. (Chongqing), Chongqing 402561, China

[*]Author for correspondence: jijialei@caas.cn (Jialei Ji), zhangyangyong@caas.cn (Yangyong Zhang)

Supplemental Materials and Methods

Plant materials and treatments

The plant materials used in this study included two cabbage inbred lines (MW and XW). For water-deficit (WD) induction experiments, MW plants were subjected to a 5-day-WD treatment in controlled conditions, with aboveground tissues (stems and leaves) and underground roots separately collected before and after treatment for subsequent RNA extraction. Both MW and XW lines served as genetic backgrounds for constructing overexpression and gene-edited lines through genetic transformation. Additionally, *Nicotiana benthamiana* plants were employed for luciferase complementation assays to verify protein-protein interactions. All plant materials were cultivated under standard growth conditions (22°C, 16/8 h photoperiod) in the greenhouse facilities of the Chinese Academy of Agricultural Sciences, Beijing. Experimental plants were maintained in nutrient-rich substrate with regular irrigation prior to specific treatments.

Water-deficit (WD) treatment and survival rate assessment

All experimental plants were cultivated from sowing under standardized conditions (including identical substrate, light, and temperature regimes) with quantitatively controlled watering to ensure consistent hydration until reaching the target developmental stage (approximately at full expansion of the 9th true leaf). WD stress was then uniformly initiated by simultaneously withholding all irrigation for a 2-week period. Following this treatment, full watering was resumed and plant responses were monitored daily for 5-7 days. Survival was determined by the restoration of leaf and stem turgor pressure (evident as regained upright posture) accompanied by new leaf emergence, whereas plants exhibiting persistent wilting and desiccation without recovery signs were classified as dead. The survival rate was calculated as the percentage of surviving plants relative to the initial cohort before WD exposure. This experiment employed three biological replicates per treatment group, each comprising 100 plants.

Vector construction and plant transformation

The full-length coding sequences of *BoCML37* (*BolC09g022750.2J*) and

BoABA2 (*BolC06g007060.2J*) were obtained from the Brassica Database (http://brassicadb.cn) and cloned into the CaMV 35S-driven binary vector pCAMBIA1302. These constructs were transformed into Agrobacterium tumefaciens GV3101 and introduced into cabbage inbred lines XW/MW via Agrobacterium-mediated transformation. Single-guide RNAs (sgRNAs) targeting conserved exonic regions of *BoCML37* and *BoABA2* were designed using the CRISPR-P 2.0 platform. The sgRNA was synthesized and cloned into the pHEE401E vector. The constructs were transformed into Agrobacterium strain GV3101 and introduced into cabbage inbred lines XW/MW. Edited lines were screened by genomic sequencing, and homozygous mutants with frameshift mutations were obtained for functional analysis. All transgenic and edited lines were propagated to the T_2 generation under controlled conditions to ensure genetic stability.

Subcellular localization

The full-length coding sequences of *BoCML37*, *BoABA2* and *BoNCED3* (*BolC01g048310.2J*) were fused in-frame to the N-terminus of the GFP/RFP tag, under the control of the CaMV 35S promoter in the pCAMBIA1302 vector. The constructed plasmids were transformed into Agrobacterium tumefaciens strain GV3101. For transient expression, Agrobacterium suspensions harboring individual constructs were infiltrated into leaves of 4-week-old *Nicotiana benthamiana* plants. BoCML37/BoNCED3-GFP was expressed alone to assess its localization, while co-localization assays were performed by co-expressing BoCML37-GFP with BoABA2-RFP. Plants were incubated at 22°C for 48–72 hr in darkness prior to observation. Fluorescence signals were captured using a confocal laser scanning microscope (Leica TCS SP8) with excitation/emission wavelengths of 488 nm/500-530 nm for GFP and 561 nm/580-620 nm for RFP.

Bimolecular fluorescence complementation assay

The *BoCML37* coding sequence was fused to the N-terminal Venus fragment (VN) in vector 62SK-VN-BoCML37, while *BoABA2* and *BoAAO3* were separately fused to the C-terminal fragment (VC) in 62SK-BoABA2-VC and 62SK-BoAAO3-VC. Construct pairs (62SK-VN-BoCML37 + 62SK-BoABA2-VC or

62SK-VN-BoCML37 + 62SK-BoAAO3-VC) were co-transformed into Agrobacterium tumefaciens GV3101, mixed at 1:1 ratio (OD₆₀₀ = 0.6), and co-infiltrated into 4-week-old tobacco leaves. After 48–72 hr dark incubation at 22°C, reconstituted YFP signals were visualized by confocal microscopy (514 nm excitation/530–560 nm emission), with empty VN/VC vectors serving as negative controls.

Yeast two-hybrid assay

Full-length *BoCML37* and *BoABA2* coding sequences were cloned into bait vector pGBKT7 and prey vector pGADT7 via EcoRI/BamHI restriction sites to generate N-terminal fusions (pGBKT7-BoCML37, pGADT7-BoABA2). Plasmid pairs were co-transformed into Saccharomyces cerevisiae strain AH109 using the lithium acetate/PEG method. Transformants were first selected on SD/-Leu/-Trp (DDO) plates at 30°C for 3–5 days. Protein interaction was then confirmed by growth and blue color development on high-stringency SD/-Ade/-His/-Leu/-Trp plates supplemented with 40 μg/mL X-α-Gal (QDO/A) after 3–7 days of incubation. Critical controls included pGBKT7-BoCML37 + empty pGADT7 (bait autoactivation check), empty pGBKT7 + pGADT7-BoABA2 (prey autoactivation check), and the positive control pair pGBKT7-p53 + pGADT7-T-antigen.

Co-immunoprecipitation assay

The physical interaction between BoCML37 and BoABA2 was confirmed by co-immunoprecipitation in *Nicotiana benthamiana* leaves. 35S::BoCML37-GFP and 35S::BoABA2-MYC constructs were co-expressed via Agrobacterium tumefaciens (GV3101) infiltration. Total proteins were extracted 48 hr post-infiltration using IP buffer (50 mM Tris-HCl pH 7.5, 150 mM NaCl, 1% NP-40, protease inhibitors). For immunoprecipitation, 1 mg protein lysate was incubated with anti-GFP monoclonal antibody (Abcam, ab290) at 4°C for 2 hr, followed by binding to Protein A/G Magnetic Beads (Thermo Scientific) for 1 hr. After five washes with IP buffer, bound proteins were eluted in SDS loading buffer and analyzed by Western blot with anti-MYC (1:5000, Sigma-Aldrich, M4439) to detect co-precipitated BoABA2-MYC, while anti-GFP (1:5000, Abcam, ab290) verified BoCML37-GFP pulldown efficiency.

Controls included single-tag expressions with empty vectors to exclude non-specific binding.

Pull-down assay

BoCML37 was cloned into pMAL-c5X to express MBP-BoCML37, while *BoABA2* was inserted into pET-28a(+) to produce His-BoABA2. Recombinant proteins were expressed in Escherichia coli BL21(DE3) with 0.5 mM IPTG induction (16°C, 16 hr). MBP-BoCML37 was purified using amylose resin (NEB), and His-BoABA2 was isolated via Ni-NTA agarose (Qiagen). For binding, 5 μg MBP-BoCML37 bound to amylose beads was incubated with 10 μg His-BoABA2 in interaction buffer (20 mM Tris-HCl pH 7.4, 200 mM NaCl, 1 mM EDTA, 1 mM DTT) at 4°C for 2 hr. After five washes, bound complexes were eluted in SDS buffer, resolved by 12% SDS-PAGE, and immunoblotted with anti-His antibody (1:5000, Abcam, ab18184) to detect His-BoABA2 retention, with anti-MBP antibody (1:4000, NEB, E8032S) verifying bait protein loading. MBP-tagged empty vector served as negative control.

Luciferase complementation assay

BoCML37 and *BoABA2* coding sequences were cloned into split-luciferase vectors pCAMBIA1300-nLUC and pCAMBIA1300-cLUC respectively. The constructs were co-expressed in *Nicotiana benthamiana* leaves via Agrobacterium-mediated transient expression. After 48 hours, leaf discs were placed on 0.8% agar media supplemented with 10 mM $CaCl_2$, EGTA (pH 7.0), or no additives (control). Luciferase activity was measured after D-luciferin (1 mM) incubation using a GloMax luminometer. Data from six biological replicates were analyzed by ANOVA with Tukey's test.

RNA extraction and RT–qPCR

Cabbage inbred line 'MW' was exposed to WD stress by withholding water for 7 days in a controlled greenhouse (25°C, 16-h light). Paired shoot (stems + leaves) and root samples from fully watered controls (pre-drought) and WD-stressed plants were collected simultaneously. Total RNA was extracted using TRIzol reagent, treated with DNase I, and reverse-transcribed into cDNA. Quantitative PCR utilized SYBR Green

chemistry with specific primers (validated by melt curve analysis), normalizing expression to BoACTIN via the $2^{-\Delta\Delta CT}$ method. Three biological replicates were analyzed.

Determination of ABA content

To assess ABA dynamics under WD stress, we quantified endogenous ABA levels in wild-type, *BoCML37*-overexpressing, *BoCML37*-knockout, *BoABA2*-overexpressing, and *BoABA2*-knockout cabbage lines. Leaves were harvested from well-watered plants (0-day control) and after 5 days of WD treatment (n =6 biological replicates per genotype/condition). Approximately 100 mg fresh weight tissue was flash-frozen in liquid N_2 and stored at −80°C. ABA extraction followed a standardized methanol-based protocol: frozen tissue was homogenized in 80% methanol containing 1% acetic acid and 10 ng [$^2H_6$]-ABA internal standard, incubated at 4°C overnight, then centrifuged. Supernatants were purified using solid-phase extraction (Oasis® MCX columns). Dried extracts were reconstituted and analyzed via UHPLC-ESI-MS/MS in multiple reaction monitoring (MRM) mode. Quantification was performed by comparing analyte peak areas to the deuterated internal standard, with data normalized to tissue fresh weight.

Assay of ABA2 activity

BoABA2 enzymatic activity was determined spectrophotometrically by monitoring NAD reduction kinetics at 340 nm. Assay mixtures contained 100 mM K_2HPO_4 (pH 7.2), 100 μM NAD, and 100 μM xanthoxin (synthesized from (R)-4-hydroxy-β-cyclogeraniol according to Kuba et al., 2008) in a final volume of 1 mL. Reactions were initiated by adding either: (i) 10 μL purified BoABA2 (10 ng/μL), (ii) BoABA2 in combination with 10 μL purified BoCML37 (10 ng/μL), or (iii) BoABA2 + BoCML37 + 100 μM $CaCl_2$. After 30 min incubation at 25°C, reactions were terminated with 10 μL of 0.1 mM HCl. The reduction rate of NAD to NADH was quantified using the molar extinction coefficient of NADH, with zero-enzyme controls and three technical replicates per condition.

Stress-response indicators examination

WD responses in cabbage lines were evaluated by measuring key parameters

under well-watered conditions (0-day control) and after 5 days of WD exposure. Stomatal conductance was measured on fully expanded leaves using the portable photosynthesis system. Proline content was quantified spectrophotometrically using the ninhydrin-based assay (Abdel Latef et al., 2011). Catalase (CAT) activity was determined by monitoring the decomposition of H_2O_2 at 240 nm using the UV-visible spectrophotometer (Qiu et al., 2023). Malondialdehyde (MDA) content, as an indicator of lipid peroxidation, was measured via the thiobarbituric acid reactive substances (TBARS) assay, reading absorbance at 532 nm and 600 nm (Qiu et al., 2023). Peroxidase (POD) activity was assessed using the guaiacol method, measuring the increase in absorbance at 470 nm due to guaiacol oxidation (Qiu et al., 2023). Superoxide dismutase (SOD) activity was assayed based on the inhibition of nitroblue tetrazolium (NBT) photoreduction, monitored at 560 nm (Liu et al., 2023). Relative electrolyte leakage (REC), indicating membrane damage, was determined by measuring the electrical conductivity of leaf disc leachates before and after autoclaving using a benchtop electrical conductivity meter (Zhou et al., 1998). All analyses included 6 biological replicates per treatment.

Phylogenetic Analysis

Protein sequences of BoCML37 and BoABA2 were used as queries for BLASTP searches against the NCBI database. Homologous sequences from representative plant species were aligned using MAFFT. Phylogenetic trees were reconstructed using FastTree. Final trees were visualized and annotated in FigTree.

REFERENCES

Abdel Latef AAH, Chaoxing H. Arbuscular mycorrhizal influence on growth, photosynthetic pigments, osmotic adjustment and oxidative stress in tomato plants subjected to low temperature stress. *Acta Physiol. Plant* 2011:33:1217–1225.

Qiu P, Li J, Zhang L, Chen K, Shao J, Zheng B, Yuan H, Qi J, Yue L, Hu Q, et al. Polyethyleneimine-coated MXene quantum dots improve cotton tolerance to Verticillium dahliae by maintaining ROS homeostasis. *Nat. Commun.* 2023:14(1):

7392.

Liu C, Li C, Bing H, Zhao J, Li L, Sun P, Li T, Du D, Zhao J, Wang X, Xiang W. Integrated Physiological, Transcriptomic, and Metabolomic Analysis Reveals the Mechanism of Guvermectin Promoting Seed Germination in Direct-Seeded Rice under Chilling Stress. *J. Agric. Food Chem.* 2023:71(19):7348–7358.

Zhou W, Leul M. Uniconazole-induced alleviation of freezing injury in relation to changes in hormonal balance, enzyme activities and lipid peroxidation in winter rape. *Plant Growth Regul.* 1998:26:41–47.

Kuba M, Furuichi N and Katsumura S. Stereocontrolled syntheses of carotenoid oxidativemetabolites, (-)-loliolide, (-)-xanthoxin, and their stereoisomers. *Chem. Lett.* 2008:31:1248–1249.

Supplemental Table S1. The primers used in this study.

Assay	Primer Name	Primer Sequence (5'-3')
RT-qPCR	BolC08g009210.2J_CML2-F	AGGGATACAGACCTATACCAGGC
	BolC08g009210.2J_CML2-R	CAACGCACACTGCCTACGA
	BolC04g042130.2J_CML2-F	ATGGACAGTGGAGAATTAAGTAGGG
	BolC04g042130.2J_CML2-R	CGACCTCAGTTCTTCACCTGTGATA
	BolC05g058230.2J_CML3-F	CAAACAGAGCTTTCCCGGATAT
	BolC05g058230.2J_CML3-R	ACACGTTGAACGCCTCTATGAT
	BolC03g039010.2J_CML3-F	CGTTGGAATCTACATGCCTGAC
	BolC03g039010.2J_CML3-R	CGTCTCGGTTCTGATCAAAGACA
	BolC01g053730.2J_CML3-F	ATCCCAGAGCAAGAGCTGG
	BolC01g053730.2J_CML3-R	TCATCCTCTTGCATTCATCTAGC
	BolC08g042820.2J_CML4-F	ACACCGACGACAAGATCACAC
	BolC08g042820.2J_CML4-R	CAACTATCGAACCGTAAAGAGAC
	BolC04g066240.2J_CML5-F	TGGTGGTTTTAGCTGCAGTAATAAC
	BolC04g066240.2J_CML5-R	GTCTATGTCCACGCACCCAT
	BolC09g002620.2J_CML6-F	ATGGACTCCACGGAGCT
	BolC09g002620.2J_CML6-R	AACTCTTCGACGTCAACGCAA
	BolC08g002130.2J_CML7-F	ATTCAACAGAGCTCAAACGCG
	BolC08g002130.2J_CML7-R	CCGTCGCCATTCACGTCTA
	BolC08g058420.2J_CML7-F	AGATGTGAATCCCTAGGCTTCGTAA
	BolC08g058420.2J_CML7-R	ACCCTACCATCACCGTCAA
	BolC06g016640.2J_CML8-F	AGATCATTGGGTTCAAAGAGGCT
	BolC06g016640.2J_CML8-R	AGCTCCTCTTCTGCATCACTTTC
	BolC04g040370.2J_CML9-F	ATGGCCGATGCTTTCACG
	BolC04g040370.2J_CML9-R	CGTTGCCAAAGATGTCGACA
	BolC01g039760.2J_CML11-F	ACTGCTGATGAGCTTGCC
	BolC01g039760.2J_CML11-R	TGGCCGAGATGTAGCCATT
	BolC05g039380.2J_CML11-F	GGATGAGACAAAACAACAACTGGA
	BolC05g039380.2J_CML11-R	CTGGATTTTGCTGCCCATGAGG
	BolC05g010520.2J_CML13-F	ATGGGAAAGGACGGCCTGA
	BolC05g010520.2J_CML13-R	GTTAGGCTTTCGGAGGCGGT
	BolC08g023920.2J_CML13-F	TCGCTCAAGCCATCCTTGC
	BolC08g023920.2J_CML13-R	CCATCAGATCGAGGAACCTCTC
	BolC08g054790.2J_CML13-F	ACTCCGAAAACCTAACGGCTCCG
	BolC08g054790.2J_CML13-R	TAGCAACCATCCTGGCAATGA
	BolC05g016150.2J_CML15-F	CATATTCAACCGGTTCGACATGG
	BolC05g016150.2J_CML15-R	TACAAGCTCATCAAACTCGACGA
	BolC08g050860.2J_CML15-F	ATCGGTAACAATACCGAACATC
	BolC08g050860.2J_CML15-R	GCGGATTTTGCCATTATAGAGGCA
	BolC09g028960.2J_CML16-F	TGACTTATCGCGAGTTGACG
	BolC09g028960.2J_CML16-R	AAGCAGCCGTTAATCCAAGA
	BolC05g038170.2J_CML17-F	ACAAAGACGGAAGCTTAACACAG

	BolC05g038170.2J_CML17-R	CCGTCAGTATCGAATATCCTGAA
	BolC01g056570.2J_CML18-F	GGAAGCTTGACGGAGCTAGA
	BolC01g056570.2J_CML18-R	GTCAACGCGTGTCCTAGCTT
	BolC01g001920.2J_CML19-F	AGCAGCACAGTTCAGAAGAGG
	BolC01g001920.2J_CML19-R	ACCCGAGAGACCTCATAGCA
	BolC01g028360.2J_CML20-F	GTGGACAAGGATGGAAGTGG
	BolC01g028360.2J_CML20-R	GGTCACGGTCAGCTTCTT
	BolC01g021130.2J_CML21-F	CACATTCGTGTTCCTGGATG
	BolC01g021130.2J_CML21-R	CTGCGTAAATGCGAACAAGA
	BolC07g052800.2J_CML21-F	ATGGGAAGTGCGGTGAC
	BolC07g052800.2J_CML21-R	TCAATCGACCCATTAGAATCC
	BolC07g013000.2J_CML22-F	TGTAAACTCGGAGAGCAACAAG
	BolC07g013000.2J_CML22-R	TCAGCTCCTCCATATCAATCGTA
	BolC01g038010.2J_CML22-F	TGCTAAGCTCGCACGGA
	BolC01g038010.2J_CML22-R	GCTCCAGTTCCACTAAGCAT
	BolC06g037530.2J_CML23-F	GAGCAGTATTCCAGCGCTTC
	BolC06g037530.2J_CML23-R	CGTCGAGATCGAACTCCTTC
	BolC04g047650.2J_CML24-F	ATGTCGAAGAGCAACGGAG
	BolC04g047650.2J_CML24-R	GGTTCACTCCGATCTGGAAA
	BolC03g068650.2J_CML25-F	CAGACTCTCCCTTCCTGCAC
	BolC03g068650.2J_CML25-R	TGTTATGGCCTTCTGCAACA
	BolC06g045070.2J_CML26-F	AAATCCATGGGAACGTCGTA
	BolC06g045070.2J_CML26-R	CTTCGACGGAACACGTCATA
	BolC05g015850.2J_CML27-F	GTAACCCTGTAACCGCCAAA
	BolC05g015850.2J_CML27-R	CTCCGTCTCGGTGTAGGAAG
	BolC08g026950.2J_CML27-F	GGGGACTTCATACACGGAGA
	BolC08g026950.2J_CML27-R	CTCAGAGGCGGAGATGAGTC
	BolC03g036600.2J_CML28-F	AAGAGCTGAGCACGATCGTA
	BolC03g036600.2J_CML28-R	CGCTTAATGTCGTCGTGAGTGA
	BolC05g063570.2J_CML28-F	GAAAGCAGAGCACGACCGTG
	BolC05g063570.2J_CML28-R	CGCTTAATGTCATCGTGTGTGG
	BolC09g011890.2J_CML30-F	CTTCCTTGAGCTGCAATACAAGC
	BolC09g011890.2J_CML30-R	ACCACCTTGTACTCAGTCTGAGAG
	BolC03g001630.2J_CML32-F	GTGGTTGGCGAAAAAGAAGA
	BolC03g001630.2J_CML32-R	CATGCTAGCACATTCCTCCA
	BolC06g003890.2J_CML34-F	GACAAGAACAACGACGGCA
	BolC06g003890.2J_CML34-R	GCTGTTACCTTTCCGTCACC
	BolC04g003160.2J_CML35-F	AGCTGGTCGAAGCGTTTAAG
	BolC04g003160.2J_CML35-R	TCCTCCGTCTCTACACACCC
	BolC04g065140.2J_CML35-F	AGATCCGAACCTTCGTCCTT
	BolC04g065140.2J_CML35-R	GCTTAAACGCTTGGATCAGC
	BolC09g022750.2J_CML37-F	AACATCAAGAAATCTCCCTTGGC
	BolC09g022750.2J_CML37-R	AGATTTTCCCGTCCGAGTTT

	BolC06g048130.2J_CML38-F	TCGTGAAGTTCTGTCGGAAA
	BolC06g048130.2J_CML38-R	CCGTCTCTATTTGCGTCCAT
	BolC06g048120.2J_CML39-F	ATGAAGAACACTCAACGTCAG
	BolC06g048120.2J_CML39-R	TGCTCTCCTAACGTCTTGAAACT
	BolC07g044310.2J_CML41-F	GCTTAAACTCTCCAAGAAGCAACAC
	BolC07g044310.2J_CML41-R	AAAGACCCATCAGCGTCAGT
	BolC07g048880.2J_CML42-F	ATCATCCTTCAGGCTTCGAAG
	BolC07g048880.2J_CML42-R	GGCTGAATGTAGGACTCCACC
	BolC03g077040.2J_CML42-F	CGAGCAACGGTGAGAAGAACAAG
	BolC03g077040.2J_CML42-R	GGTCGGAGAGATTAGCGTTGAGG
	BolC07g026470.2J_CML43-F	TGGAGATCACCATTAACGAGAAGA
	BolC07g026470.2J_CML43-R	TTTGAGGTCCGAGATATCCG
	BolC05g019930.2J_CML44-F	ATACTAGAGAGACTCGGTTGGTCC
	BolC05g019930.2J_CML44-R	TCCGTTCACGTCAAACACAT
	BolC09g003900.2J_CML46-F	AACACCACCAACGTGTTGAA
	BolC09g003900.2J_CML46-R	TACCGTTCTTGAAGCCCATC
	BolC07g036430.2J_CML46-F	ACGTGTGAGGAACCAAGACC
	BolC07g036430.2J_CML46-R	TGTAGCGTCGATGAAGCCG
	BolC04g044950.2J_CML46-F	ACTTCTCGCCGACTCAGAAA
	BolC04g044950.2J_CML46-R	CTTTGTTTCCGTCGAACGAT
	BolC03g067030.2J_CML47-F	CTCACCATTGCCACCTTCTT
	BolC03g067030.2J_CML47-R	CTTTTCTTGCGACTGGAAGC
	BolC03g029110.2J_CML48-F	GAATCTCTGCTACGGCTTGG
	BolC03g029110.2J_CML48-R	GCTGGAGAACAGAGGATGGA
	BolC01g052390.2J_CML49-F	GGATCCGAACATCGTGACTT
	BolC01g052390.2J_CML49-R	CTTTCGGTCCGATCTTTCTG
	BolC09g067840.2J_CML50-F	GGAATCAACCTCCCCCTCCT
	BolC09g067840.2J_CML50-R	TCTCATTCGGCGGGTTGAA
	BolC03g001730.2J_CML50-F	GTATTCAGGGCACGGTGGAGT
	BolC03g001730.2J_CML50-R	TCTCTGTTGACACGAGGAGAG
	BolC06g007060.2J_BoABA2-F	TCGGAGGAGTCAGCTTGTTT
	BolC06g007060.2J_BoABA2-R	TCACGTTGACGTTGAAGACC
	BolC01g020140.2J_BCH-F	CTAACATTCAAACCACTCCACCG
	BolC01g020140.2J_BCH-R	AGCTTGTTTTCTCGGGACTCT
	BolC02g020040.2J_BCH-F	ATGTCCCTTACCTTCGAAAAGTC
	BolC02g020040.2J_BCH-R	TAAGAGGTGGAACCCTTGTTGTA
	BolC07g023830.2J_ZEP-F	TCCCTGTGACTCGGGTGATTA
	BolC07g023830.2J_ZEP-R	TGAGTAAGTAGCTTCGCTTCGAC
	BolC09g010400.2J_ZEP-F	GAGTTCAACTCCGATTTGCT
	BolC09g010400.2J_ZEP-R	CGACGGCTCCTTGATTTTCC
	BolC05g052010.2J_NCED3-F	ACCTTAAATACTTCCGCTTCTCG
	BolC05g052010.2J_NCED3-R	AATTCCGAATCTTGCGACCTT
	BolC01g048310.2J_NCED3-F	CTTGCTCTTTGCCTATGACAAGT

	BolC01g048310.2J_NCED3-F	CTGTTTAGTGTCGGAGTCTTTGG
	BoIC04g053960.2J_AAO3-F	GCTATGGGTTTGCTTAGAGGAAT
	BoIC04g053960.2J_AAO3-R	GAGACTCTGTGGGATCTACATGG
Gene cloning	BoCML37-F	ATGACTCTTGCAAACATCAAGAAATCTC
	BoCML37-R	TCATCGCATCATCAGAAGAAACTC
	BoABA2-F	ATGAACAACAGGCTTTTGGGT
	BoABA2-R	TCATCTAAAAACTTTAAAGGAGTGG
	BoAAO3-F	ATGCTATCAAAATACGATCCAGAGT
	BoAAO3-R	TTATTTCCCACAGATCTTCCCTTC
	BoNCED3-F	ATGGCTTCTTTCACGGCGACTAC
	BoNCED3-R	TTACACCTGCTTCGCCAAGTCACTG

Supplemental Table S2. The survival rate of the transgenic lines.

Values followed by the same superscript letters indicate no significant difference at $P = 0.05$, based on the Duncan's test.

	**Survival rate (%) before water-deficit treatment**	**Survival rate (%) after water-deficit treatment**
WT (MW)	100±0.00	43.33±3.06 [b]
WT (XW)	100±0.00	43.00±4.00 [b]
Empty Vector (MW)	100±0.00	42.86±4.68 [b]
Empty Vector (XW)	100±0.00	43.17±3.15 [b]
CML-OE1	100±0.00	78.67±7.02 [a]
CML-OE2	100±0.00	80.00±9.16 [a]
CML-OE3	100±0.00	86.67±3.06 [a]
cml-CR1	100±0.00	10.33±3.22 [c]
cml-CR2	100±0.00	9.67±3.21 [c]
ABA-OE1	100±0.00	80.00±7.21 [a]
ABA-OE2	100±0.00	81.33±7.50 [a]
aba-CR1	100±0.00	1.33±0.58 [d]
aba-CR2	100±0.00	1.33±1.53 [d]
aba-CR3	100±0.00	1.00±1.00 [d]

Supplemental Table S3. The reproductive capacity of the transgenic lines.

For each line under well-watered condition, seeds produced by manual self-pollination were counted in 100 siliques. Values followed by the same superscript letters indicate no significant difference at P = 0.05, based on the Duncan's test.

	**Number of seeds per silique (mean ± SD)**
WT (MW)	19.17 ± 2.14^{a}
WT (XW)	19.86 ± 3.50^{a}
Empty Vector (MW)	20.33 ± 4.37^{a}
Empty Vector (XW)	19.58 ± 4.22^{a}
CML-OE1	20.56 ± 4.78^{a}
CML-OE2	19.88 ± 3.22^{a}
CML-OE3	19.15 ± 3.67^{a}
cml-CR1	19.67 ± 2.53^{a}
cml-CR2	21.33 ± 5.21^{a}
ABA-OE1	20.37 ± 4.67^{a}
ABA-OE2	18.97 ± 4.21^{a}
aba-CR1	3.57 ± 2.35^{b}
aba-CR2	2.97 ± 1.67^{b}
aba-CR3	3.33 ± 2.15^{b}

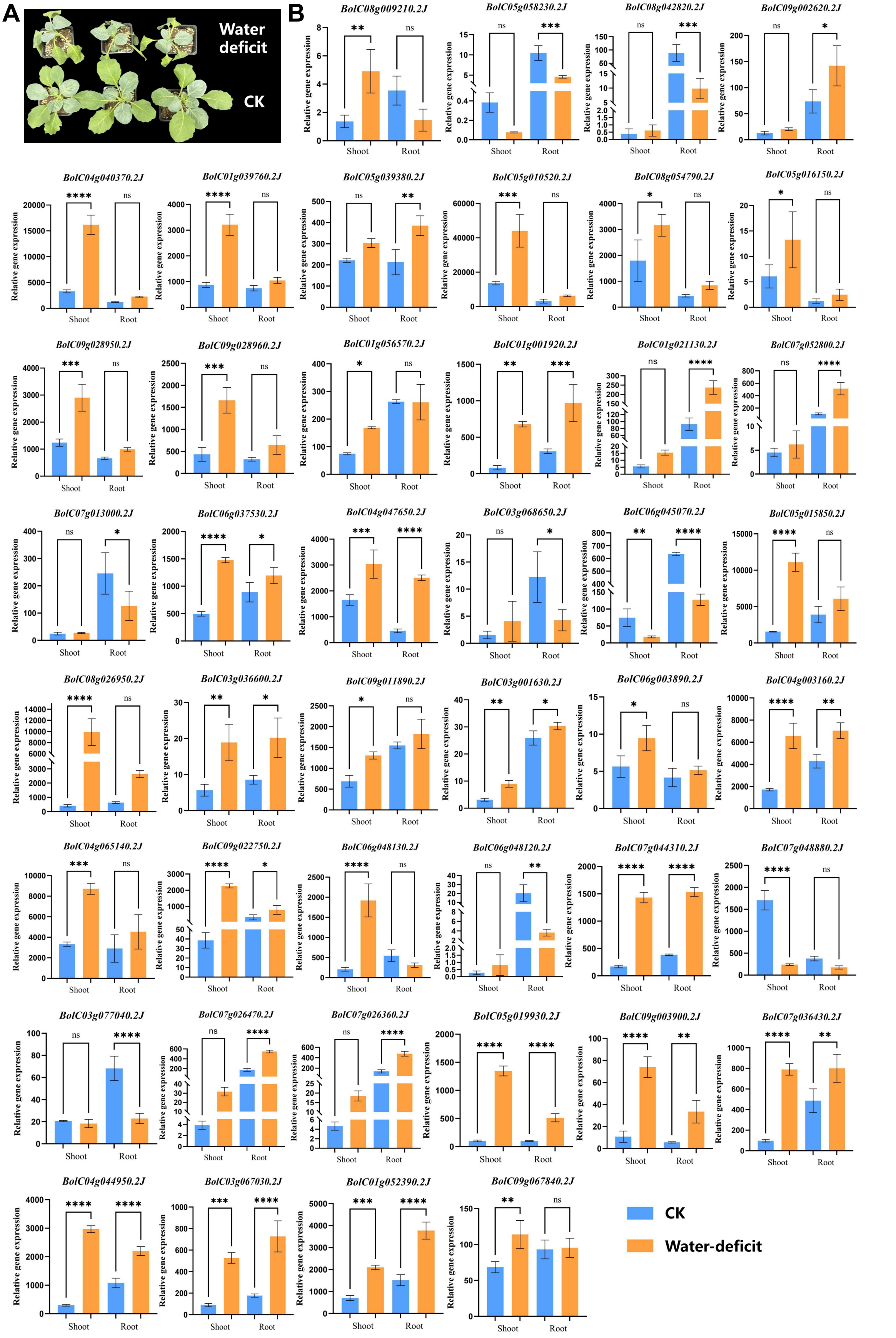

Supplemental Figure S1. Transcript abundance analysis of BoCML genes pre- and post-water-deficit treatment. (A) Phenotype of 30-day-old plants before and after a 5-day water-deficit treatment. Images were digitally extracted for comparison. (B) Transcript abundance analysis of BoCML genes. Values are means ± SE (n=3). Statistical significance between the well-watered control (CK) and water-deficit condition was assessed by Student's *t*-test. * $P < 0.05$, ** $P < 0.01$, *** $P < 0.001$, **** $P < 0.0001$; ns, non-significant, $p > 0.05$.

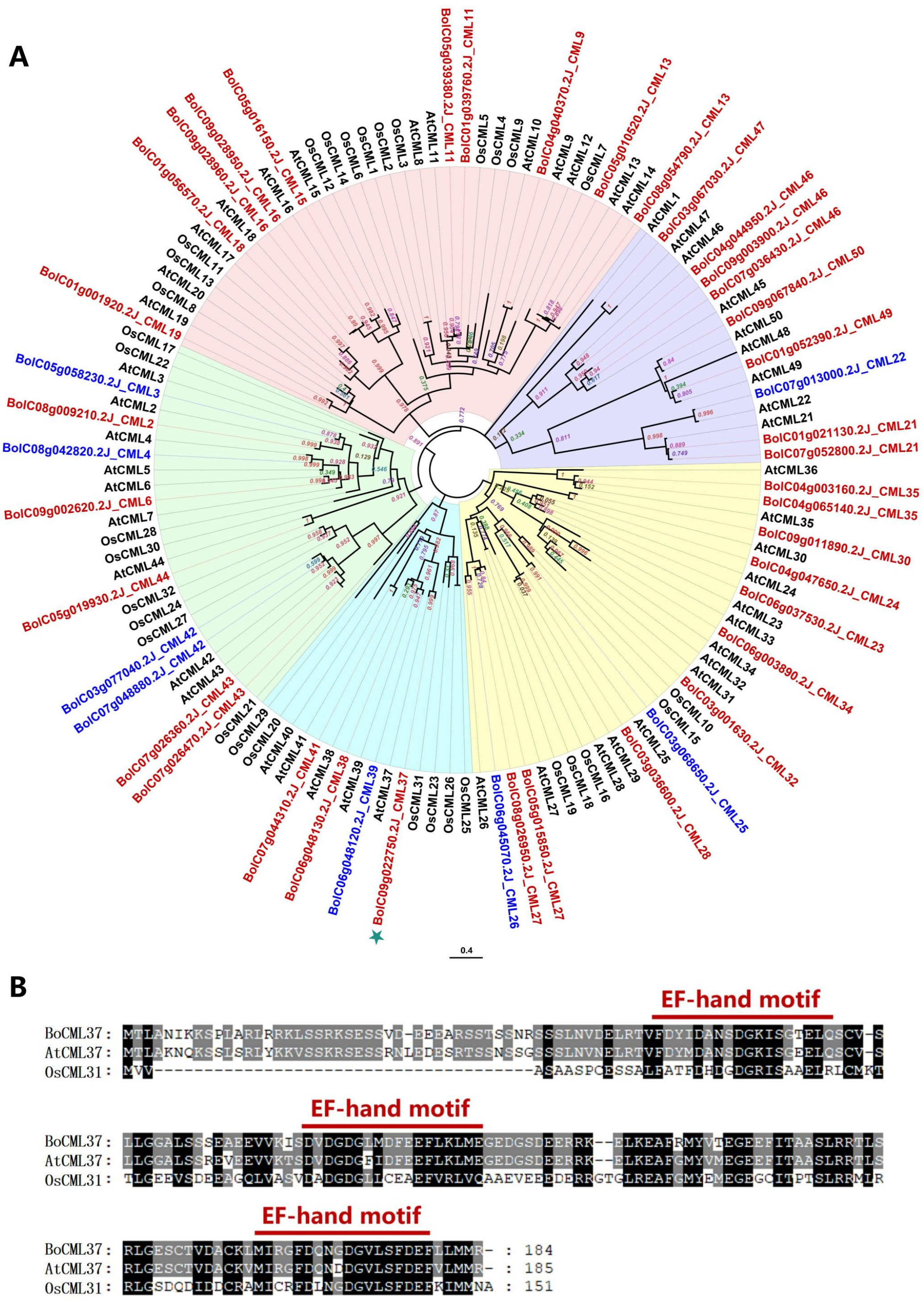

Supplemental Figure S2. Phylogenetic analysis of BoCML proteins and multiple sequence alignment of BoCML37 homologous proteins.

(A) Phylogenetic tree of CML proteins from cabbage, *Arabidopsis thaliana*, and rice. BoCML transcript abundance up-regulated in response to water-deficit stress are in red font, while those down-regulated are in blue font. The scale bar represents an evolutionary distance of 0.4 substitutions per site. (B) Multiple sequence alignment of the protein sequences of BoCML37, AtCML37, and OsCML31.

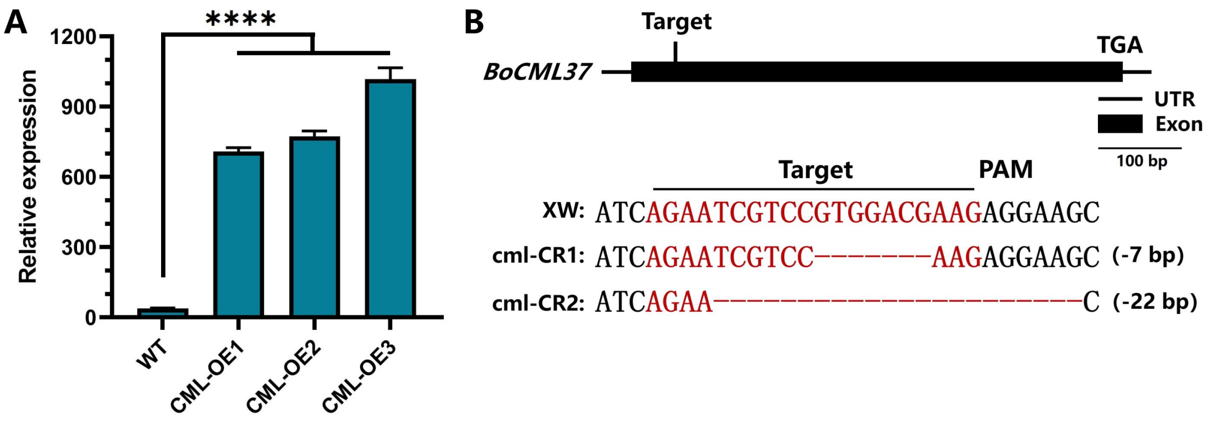

Supplemental Figure S3. Transcript abundance analysis of *BoCML37* in overexpression lines and variant analysis of *BoCML37* in gene-edited lines.
(A) Relative expression levels of the *BoCML37* gene in wild-type (WT) and three overexpression lines (CML-OE1, CML-OE2, CML-OE3). Statistical significance between the WT and the overexpression line was assessed by Student's *t*-test. **** $P < 0.0001$. (B) Genomic structure of the *BoCML37* gene, CRISPR target site sequence, and types of edits in two gene-edited lines (cml-CR1, cml-CR2).

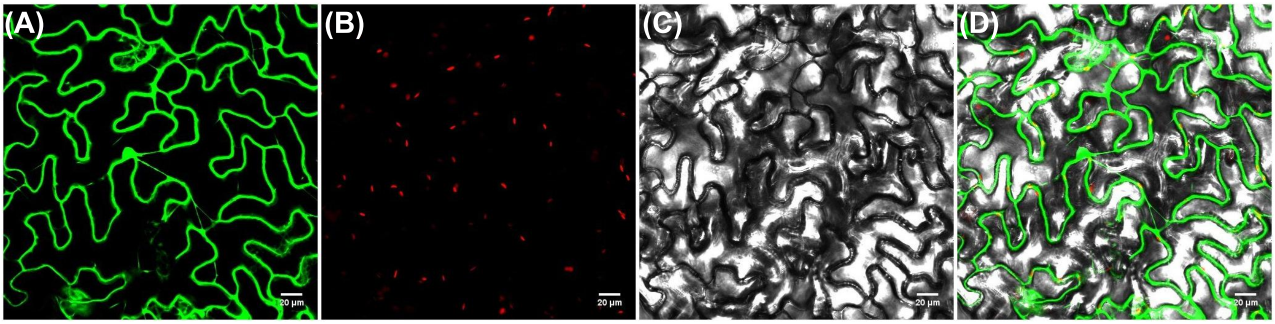

Supplemental Figure S4. Subcellular localization of the BoCML37 protein in *Nicotiana benthamiana* leaves. Confocal microscopy of leaf epidermal cells transiently expressing 35S::BoCML37-GFP. (A) BoCML37-GFP fluorescence signal. (B) Chloroplast autofluorescence. (C) Bright-field image. (D) Merged channels (A-C). Scale bar = 20 μm.

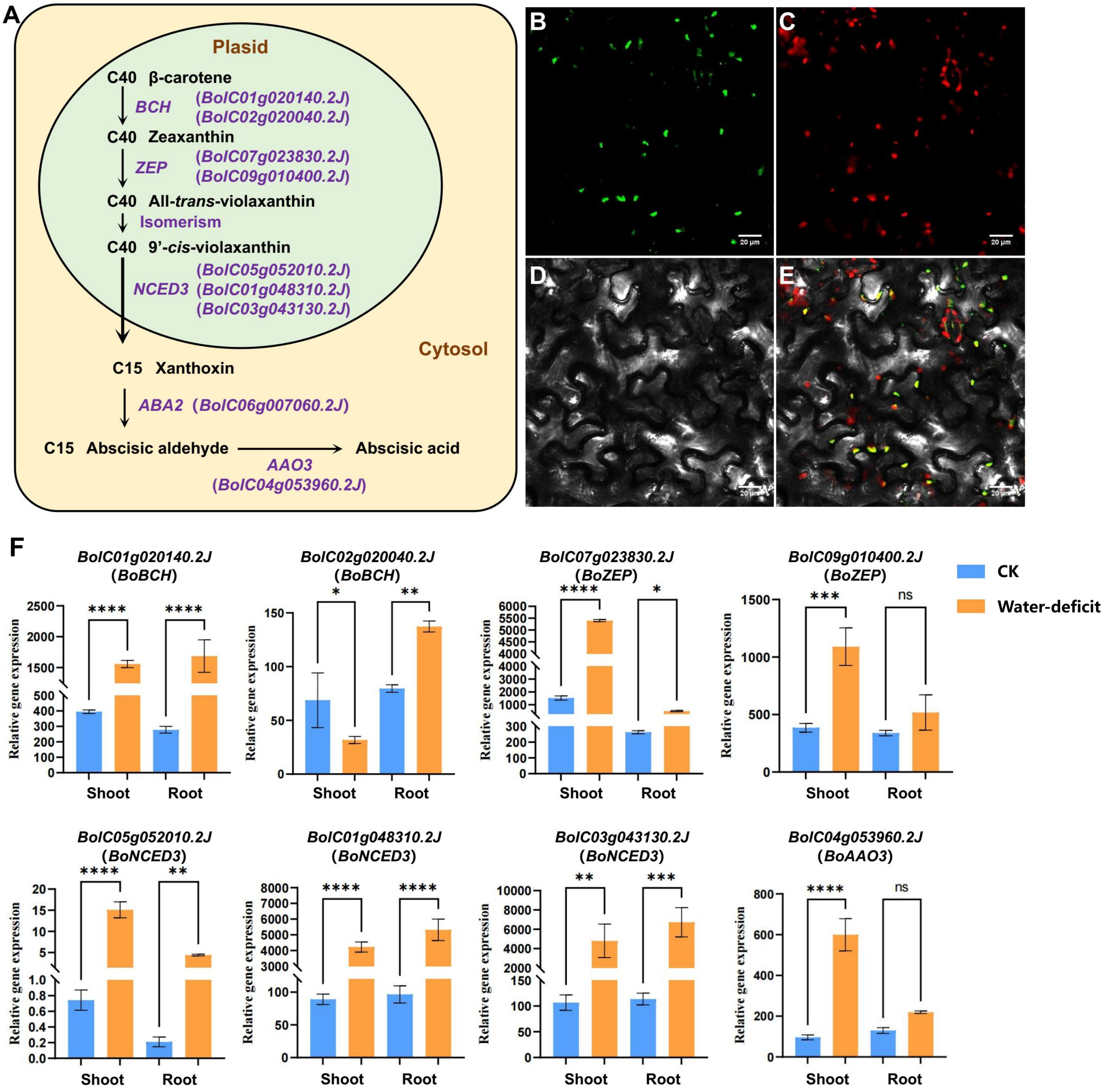

Supplemental Figure S5. Transcript abundance analysis of ABA biosynthesis-related genes and subcellular localization of BoNCED3 (BolC01g048310.2J). (A) ABA biosynthesis pathway diagram. (B) Subcellular localization of BoNCED3-GFP in *Nicotiana benthamiana*. (C) Red fluorescent signal in chloroplasts of *N. benthamiana*. (D) Bright field of *N. benthamiana* cells. (E) Superposition of B-C. (F) ABA biosynthesis genes transcript abundance analysis before and after water-deficit treatment. Values are means ± SE (n=3). Statistical significance between the control (CK, normal water) and water-deficit condition was assessed by Student's t-test. * $P < 0.05$, ** $P < 0.01$, *** $P < 0.001$, **** $P < 0.0001$; ns, non-significant, $p > 0.05$. Scale bar = 20 μm.

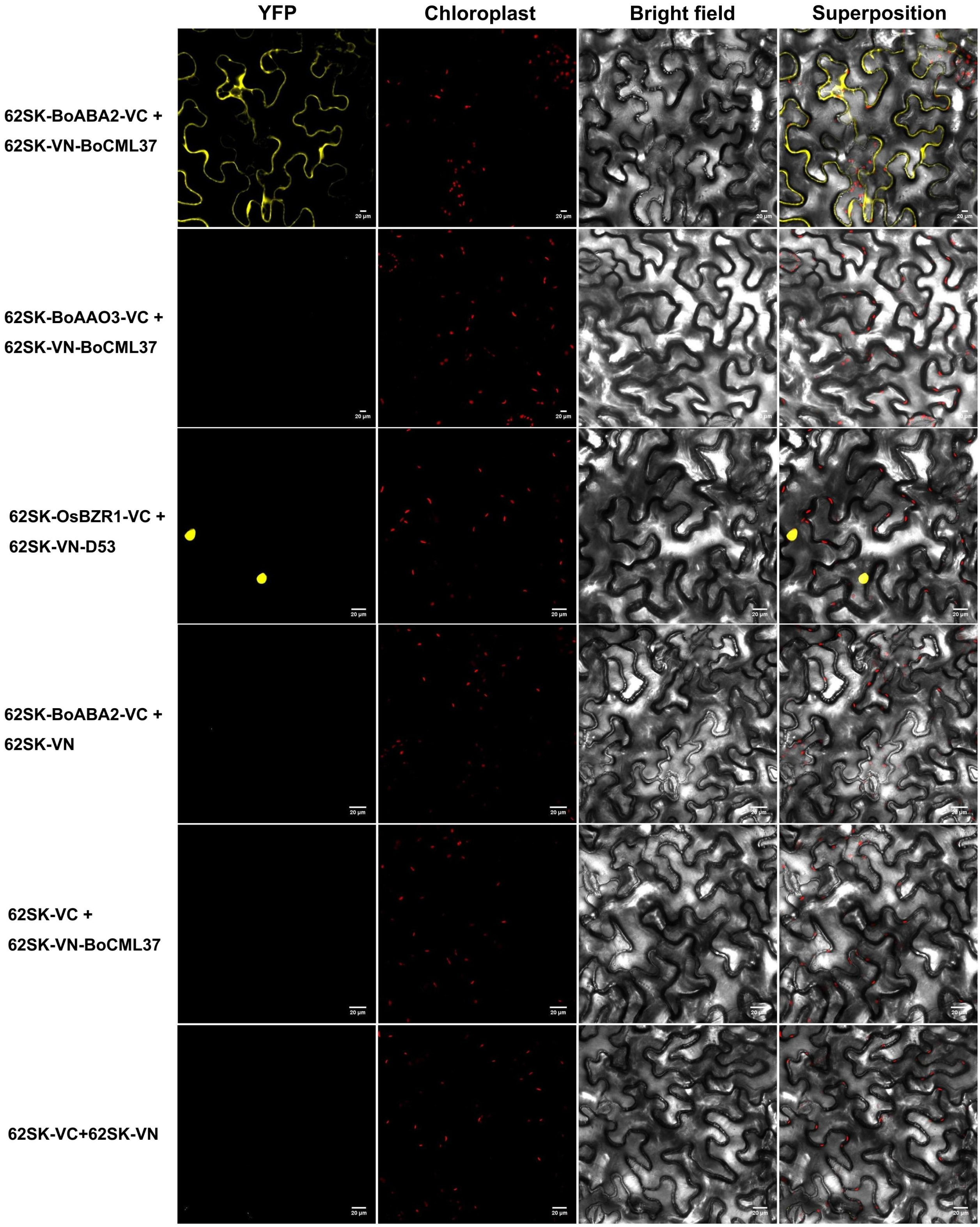

Supplemental Figure S6. Analysis of the interaction between BoCML37 and BoABA2/BoAAO3 by Bimolecular Fluorescence Complementation (BiFC) in *Nicotiana benthamiana*. 62SK-OsBZR1-VC + 62SK-VN-D53 is the positive control. GenBank/EMBL accession numbers: OsBZR1, Os07g39220; DWARF53 (D53), Os11g01330. 62SK-BoABA2-VC + 62SK-VN, 62SK-VC + 62SK-VN-BoCML37, and 62SK-VC + 62SK-VN are the negative controls. Images in row 1 are the same as those in Figure 1D. Scale bar = 20 μm.

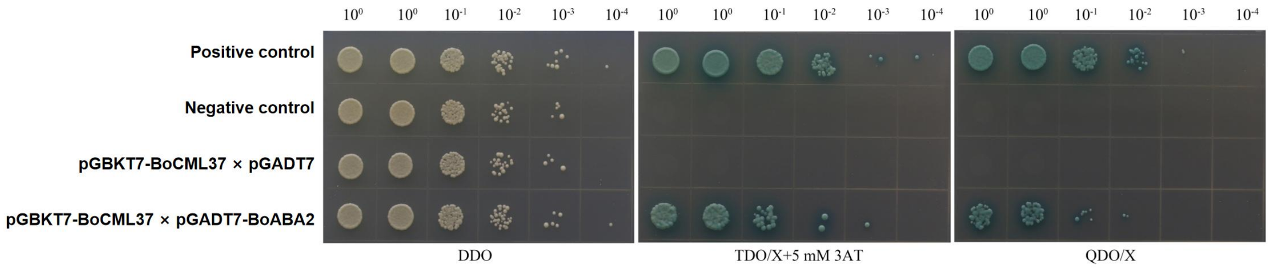

Supplemental Figure S7. Verification of the interaction between BoCML37 and BoABA2 by Yeast Two-Hybrid (Y2H) assay. Serial dilutions (10^0–10^{-4}) of yeast co-transformants were plated on control (DDO) and selective (TDO/X + 5 mM 3-AT, QDO/X) media. Growth on selective media indicates a specific BoCML37-BoABA2 interaction, whereas the lack of growth in controls (BoCML37 alone and negative control) confirms assay specificity.

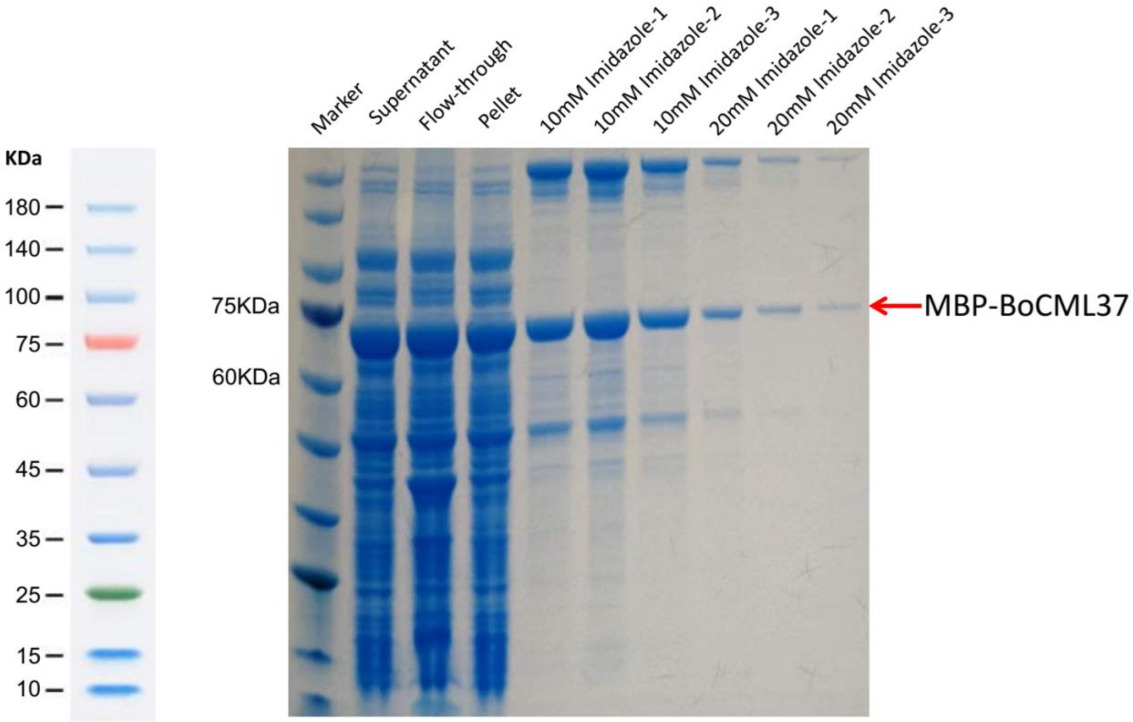

Supplemental Figure S8. SDS-PAGE of purified BoCML37 protein. The final 20 mM imidazole elution (lanes 8-10) yielded a highly purified fusion protein, consistent with the predicted size of MBP-BoCML37. The protein ladder was obtained from ABconal (Wuhan, China).

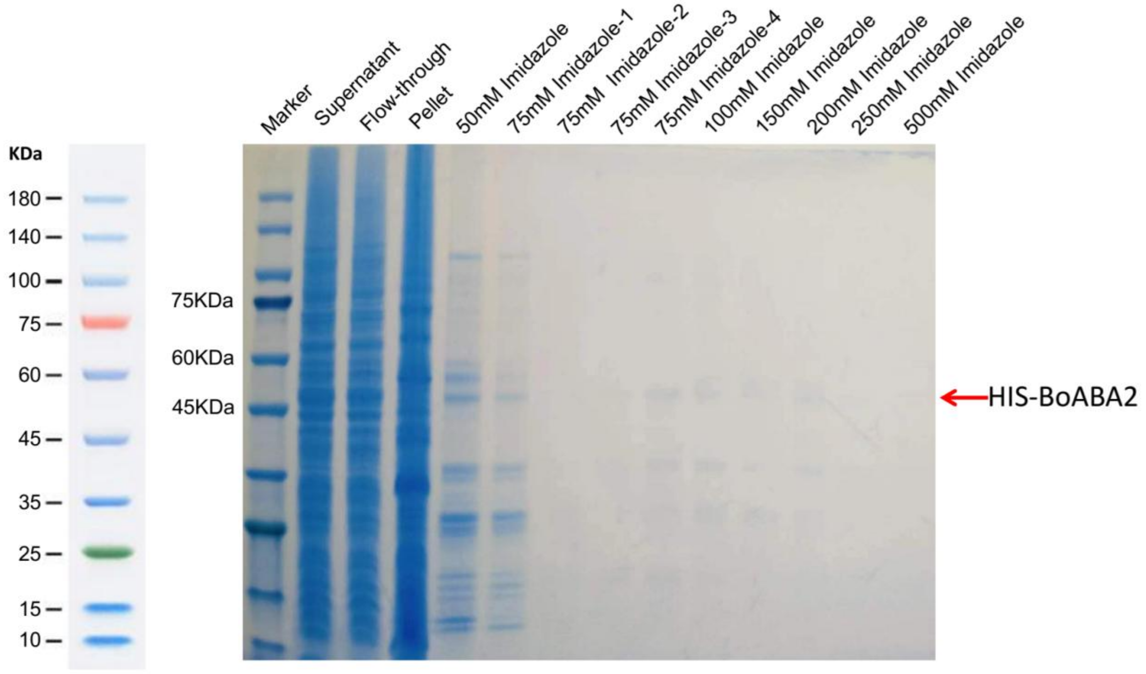

Supplemental Figure S9. SDS-PAGE of purified BoABA2 protein. A highly purified band (red arrow) is observed in the 75 mM imidazole elution, confirming the successful isolation of BoABA2 for downstream use. The protein ladder (ABconal) shown is the same as in Figure S8.

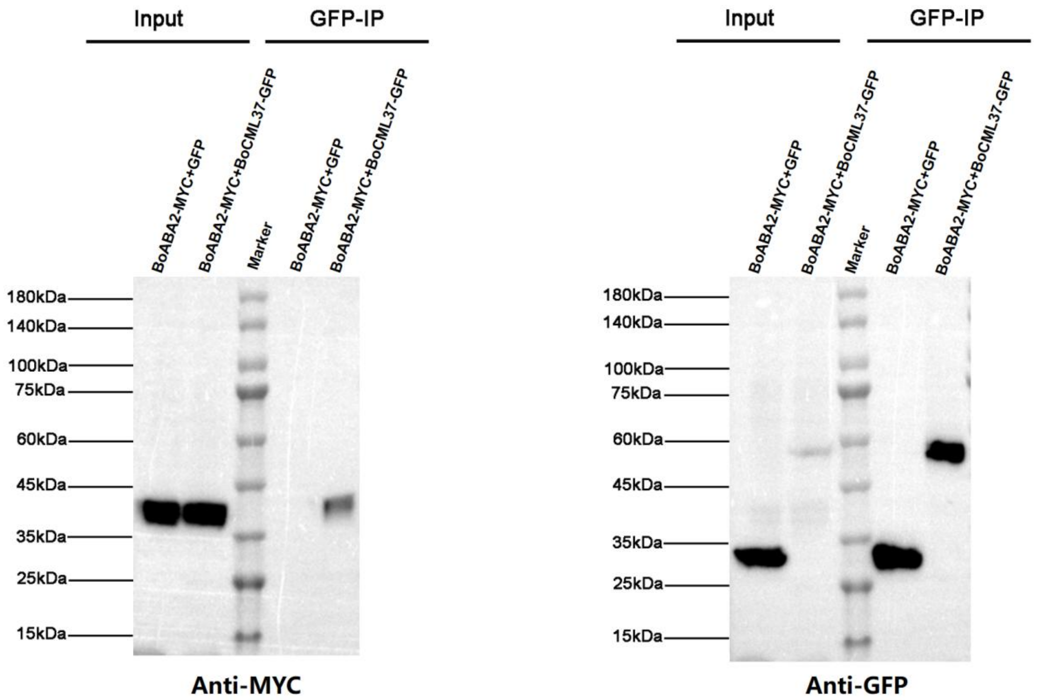

Supplemental Figure S10. Western blot of the co-immunoprecipitation (Co-IP) assay validating the interaction between BoCML37 and BoABA2. Immunoblots of proteins co-expressed in *N. benthamiana* show that BoABA2-MYC is co-precipitated specifically by an anti-GFP antibody only in the presence of BoCML37-GFP. The absence of this band in control samples confirms a specific interaction.

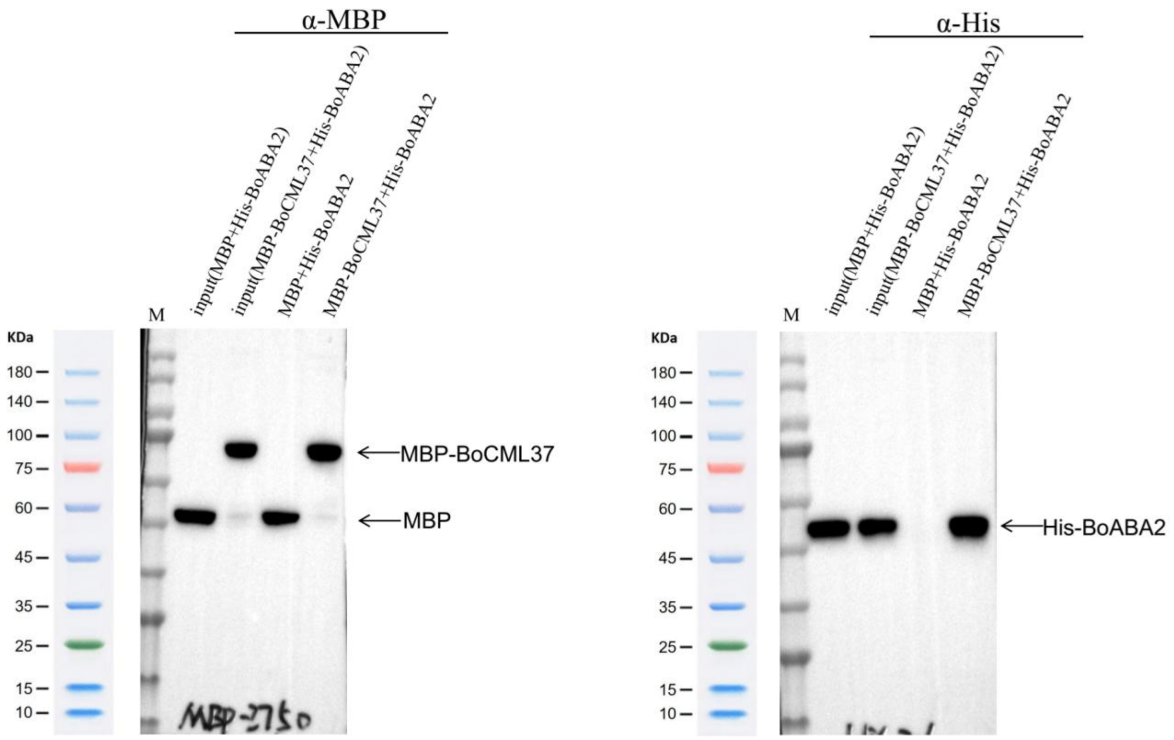

Supplemental Figure S11. Western blot of the GST pull-down assay validating the interaction between BoCML37 and BoABA2. His-BoABA2 immobilized on Ni-NTA beads specifically retains MBP-BoCML37, as visualized by Western blot. The lack of signal in control samples confirms the interaction is direct and specific. The protein ladder (ABconal) shown is the same as in Figure S8.

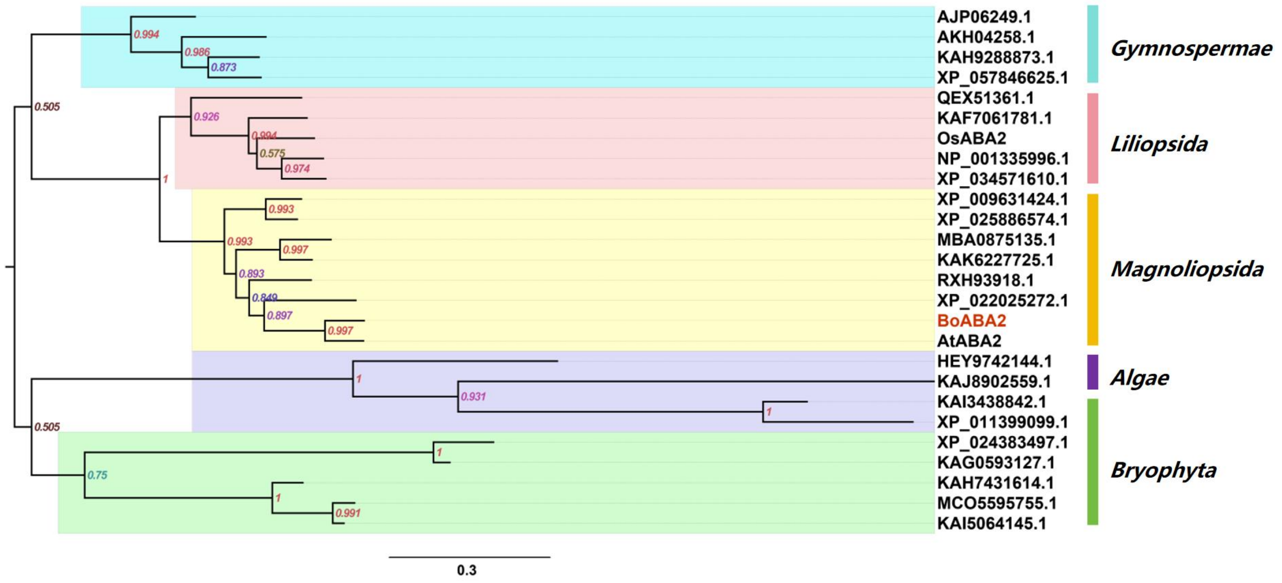

Supplemental Figure S12. Phylogenetic analysis of BoABA2.

Magnoliopsida: BoABA2 (*Brassica oleracea*), AtABA2 (*Arabidopsis thaliana*), XP_022025272.1 (*Helianthus annuus*), XP_009631424.1 (*Nicotiana tomentosiformis*), XP_025886574.1 (*Solanum lycopersicum*), KAK6227725.1 (*Theobroma cacao*), RXH93918.1 (*Malus domestica*), MBA0875135.1(*Gossypium schwendimanii*). *Liliopsida*: OsABA2 (*Oryza sativa*), KAF7061781.1 (*Triticum aestivum*), NP_001335996.1 (*Zea mays*), QEX51361.1 (*Cymbidium ensifolium*), XP_034571610.1 (*Setaria viridis*). *Gymnospermae*: AJP06249.1 (*Pinus tabuliformis*), KAH9288873.1 (*Taxus chinensis*), AKH04258.1 (*Taxus baccata*), XP_057846625.1 (*Cryptomeria japonica*). *Polypodiopsida*: MCO5595755.1 (*Adiantum nelumboides*), KAH7431614.1 (*Ceratopteris richardii*), KAI5064145.1 (*Adiantum capillus-veneris*). *Bryophyta*: XP_024383497.1 (*Physcomitrium patens*), KAG0593127.1 (*Ceratodon purpureus*), KAI3438842.1 (*Chlorella vulgaris*), XP_011399099.1 (*Auxenochlorella protothecoides*). *Algae*: KAJ8902559.1 (*Rhodosorus marinus*), HEY9742144.1 (*Coleofasciculaceae cyanobacterium*). The scale bar represents an evolutionary distance of 0.3 substitutions per site.

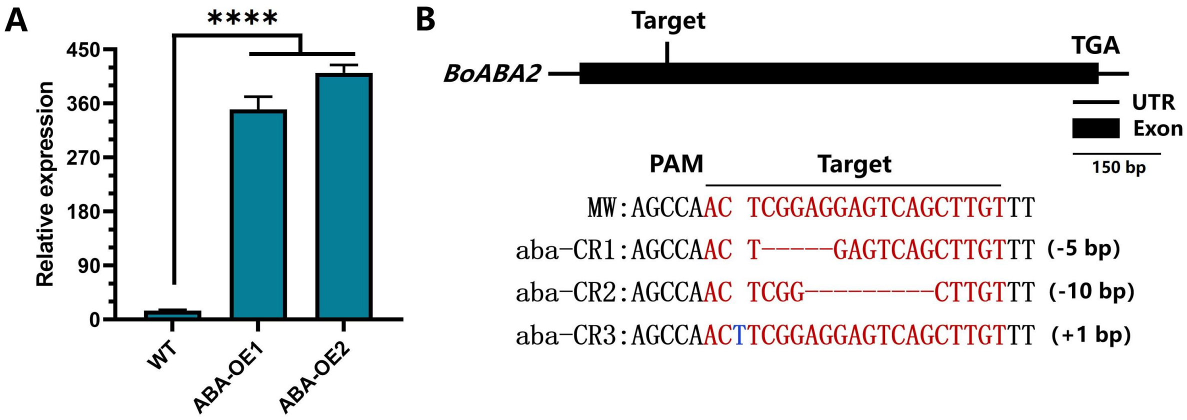

Supplemental Figure S13. Transcript abundance analysis of BoABA2 in overexpression lines and variant analysis of BoABA2 in gene-edited lines.

(A) Relative expression levels of the BoABA2 gene in wild-type (WT) and two overexpression lines (ABA-OE1, ABA-OE2). Statistical significance between the WT and the overexpression line was assessed by Student's *t*-test. **** $P < 0.0001$. (B) Genomic structure of the BoABA2 gene, CRISPR target site sequence, and types of edits in three gene-edited lines (aba-CR1, aba-CR2, aba-CR3).
